# Varicella Vaccination Alters the Chronological Trends of Herpes Zoster and Varicella

**DOI:** 10.1371/journal.pone.0077709

**Published:** 2013-10-30

**Authors:** Po-Yuan Wu, Hong-Dar Isaac Wu, Tzu-Chieh Chou, Fung-Chang Sung

**Affiliations:** 1 Department of Public Health, China Medical University, Taichung, Taiwan; 2 Department of Dermatology, China Medical University Hospital, Taichung, Taiwan; 3 School of Medicine, China Medical University, Taichung, Taiwan; 4 Department of Applied Mathematics, National Chung Hsing University, Taichung, Taiwan; 5 Management Office for Health Data, China Medical University Hospital, Taichung, Taiwan; University of Liverpool, United Kingdom

## Abstract

**Background:**

Population studies on trends of varicella and herpes zoster (HZ) associated with varicella zoster vaccination and climate is limited.

**Methods:**

This study used insurance claims data to investigate the chronological changes in incident varicella and HZ associated with varicella zoster vaccination. Poisson regression was used to estimate the occurrence of varicella associated with the occurrence of HZ and vice versa by year, season, sex, temperature, and sunny hours.

**Results:**

The varicella incidence declined from 7.14 to 0.76 per 1,000 person-years in 2000–2009, whereas the HZ incidence increased from 4.04 to 6.24 per 1,000 person-years. Females tended to have a higher risk than men for HZ (*p*<0.0001) but not varicella. The monthly mean varicella incidence was the lowest in September (160 cases) and the highest in January (425 cases), while the mean HZ incidence was lower in February (370 cases) and higher in August (470 cases). HZ was negatively associated with the incidence of varicella before and after the varicella zoster vaccination (*p*<0.001), increased 1.6% within one week post-vaccination. The effect of temperature on HZ was attenuated by 18.5% (p<0.0001) in association with vaccination. The varicella risk was positively associated with sun exposure hours, but negatively associated with temperature only before vaccination.

**Conclusions:**

The varicella vaccination is effective in varicella prevention, but the incidence of HZ increases after vaccination. HZ has a stronger association with temperature and UV than with seasonality while varicella risk associated with temperature and UV is diminished.

## Introduction

The varicella zoster virus (VZV) has a unique clinical manifestation, causing primary infections in children and latent infections as herpes zoster (HZ) mainly in adults. Weakened cellular immunity causes the reactivation of VZV, and unimmunized persons in contact with herpes zoster are at risk of varicella infections. The observational studies have shown that exposure to varicella boosts VZV-specific immunity and reduced subsequent HZ activation [1], [2]. Other studies reported that zoster is predicted to increase in unvaccinated subjects as a consequence of the reduced spreading of the virus in the population related to childhood vaccination [3]. Hence, large-scale varicella vaccination implementation may reduce the chances of boosting natural immunity, and increase the possibility of HZ infection.

Several studies have used mathematical modeling to simulate the incidence of HZ after varicella vaccinations [4]–[6]. In contrast, a French study involving 40 isolated monasteries showed that adults not exposed to VZV do not have increased HZ risk compared with the general population [7]. Similarly, a study in the United States using a large medical claims database also reported no significant difference in HZ incidence despite the varicella vaccination coverage in states [8]. Considering these conflicting results, we designed an ecological study using a national representative insurance claims dataset to investigate chronological trends of the diseases, the duality of varicella-herpes zoster incidence using a dual model, and the risk associated with varicella vaccination by the seasonality and temperature.

## Methods

### Data Source and Study Subjects

This ecological study used the National Health Insurance claims data obtained from the National Health Research Institute (NHRI) in Taiwan. This dataset consisted of the longitudinal health care information of 1 million insured individuals randomly sampled from 23 million people, allowing us to estimate the incidence and seasonality of HZ and varicella. The scrambled patient identifications were used to link files, including information on patient demographic status and health care received. The scrambled identifications safeguarded personal privacy and confidentiality. The codes of health care providers and the costs of care covered by the insurance program were also available. Diagnoses were coded using the International Classification of Diseases 9^th^ Revision of the Clinical Modification (ICD-9-CM) for 2000. Patients diagnosed with HZ and varicella (ICD code 052.X-053.XX) from January 1, 2000 to December 31, 2009 was identified from the NHRI dataset. If multiple visits were found in database, the date the patient was initially diagnosed with VZV infection was defined as the index date.

The pilot pediatric varicella immunization program in Taiwan was initiated in limited areas, before launching the nationwide comprehensive immunization program in 2004. Hence, 2000 to 2003 is the pre-vaccination period, 2004 to 2005 is the early varicella vaccination implementationperiod, and 2006 to 2009 is the full-vaccination period with an average vaccination coverage rate of pediatric varicella immunization of about 90%. All children in Taiwan are required to complete regular free vaccinations after their 12 months of age.

We also obtained 24-hour weather data from all 23 monitored stations in Taiwan courtesy of the Central Weather Bureau. The daily measured weather data from real-time monitoring stations were representative of the local population exposure to ambient temperature and sunny hours.

### Statistical Analysis

We calculated the annual incidence of rates of varicella and HZ from 2000 to 2009. Seasonality of the infections was also presented. In order to observe the association with weather, we further summarized the monthly mean incidence rates in the 10 years. Poisson regression was used to estimate the occurrence of varicella associated with the occurrence of HZ and vice versa by year, season, sex, and sunny hours. If we treated the incidence of HZ (hereafter denoted as *Z*) as an outcome variable and the incidence of varicella (denoted as *V*) as an explanatory variable, then “year” (or trend in year), “season” (or seasonality), “temperature” (weekly average), and “sunny hours” (weekly average) were considered confounding variables. These confounding variables were expressed as the vector 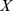 for convenience. At time “t”, we have a *primary* model to view the incidence as an outcome variable *Z*:
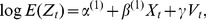
(1)where 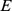
(.) is an expectation. The Poisson regression model (M1) can also measure the infection in a reverse direction, i.e., treating the incidence of varicella as an outcome and that of herpes zoster as an explanatory variable. Still, 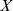 is the same confounding vector with different coefficients. We call this the *secondary* model:



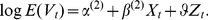
(2)The rationale behind considering a primary model plus a secondary model (i.e., considering these two models simultaneously) depends on the type of infection. The roles of the primary and secondary models are interchangeable, depending on which incidence (varicella or herpes zoster) is the main concern. We call (M1) plus (M2), with suitable and flexible time index t, a *dual model*, measured in person-weeks.

**Figure 1:**
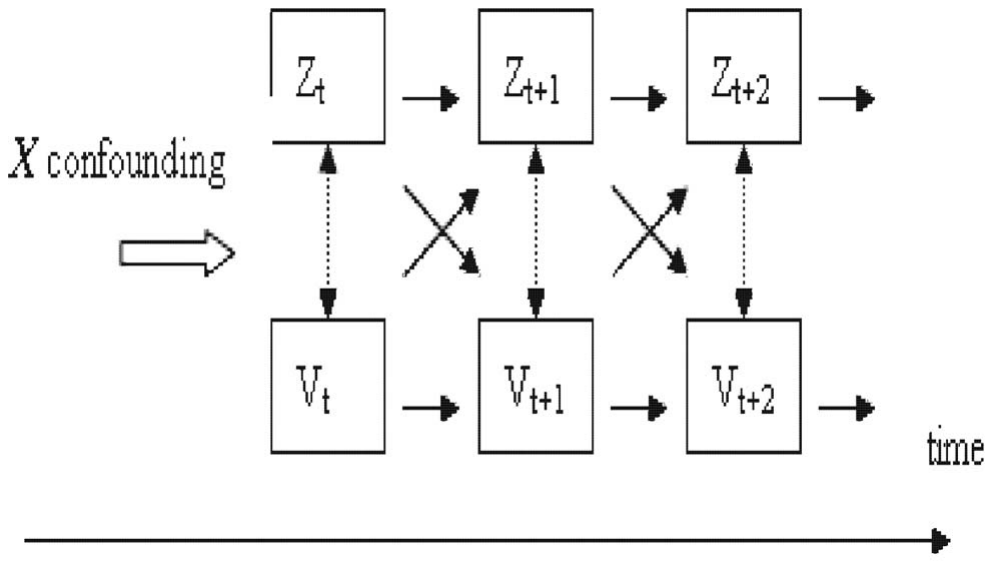
Interactive pattern between Z(herpes zoster) and V(varicella) over the time axis.

The dual model reflects the *physically* interactive pattern between *Z* and *V* over the time axis. We illustrated this idea using the diagram of Figure 1.

This model can be a complex causal structure with longitudinal explorations and data collection. It is a difficult structure to express precisely and a complex model to estimate with accuracy. We represented the *dual model* (M1+M2) simply by taking a “cross-sectional” time index, i.e. we ignored the time lag effect of Z_t-k_ on V_t_ for model M1, or that of V_t-k_ on Z_t_ for model M2, when “k” represented the lag order.

## Results

### Descriptive Epidemiology

**Figure 2:**
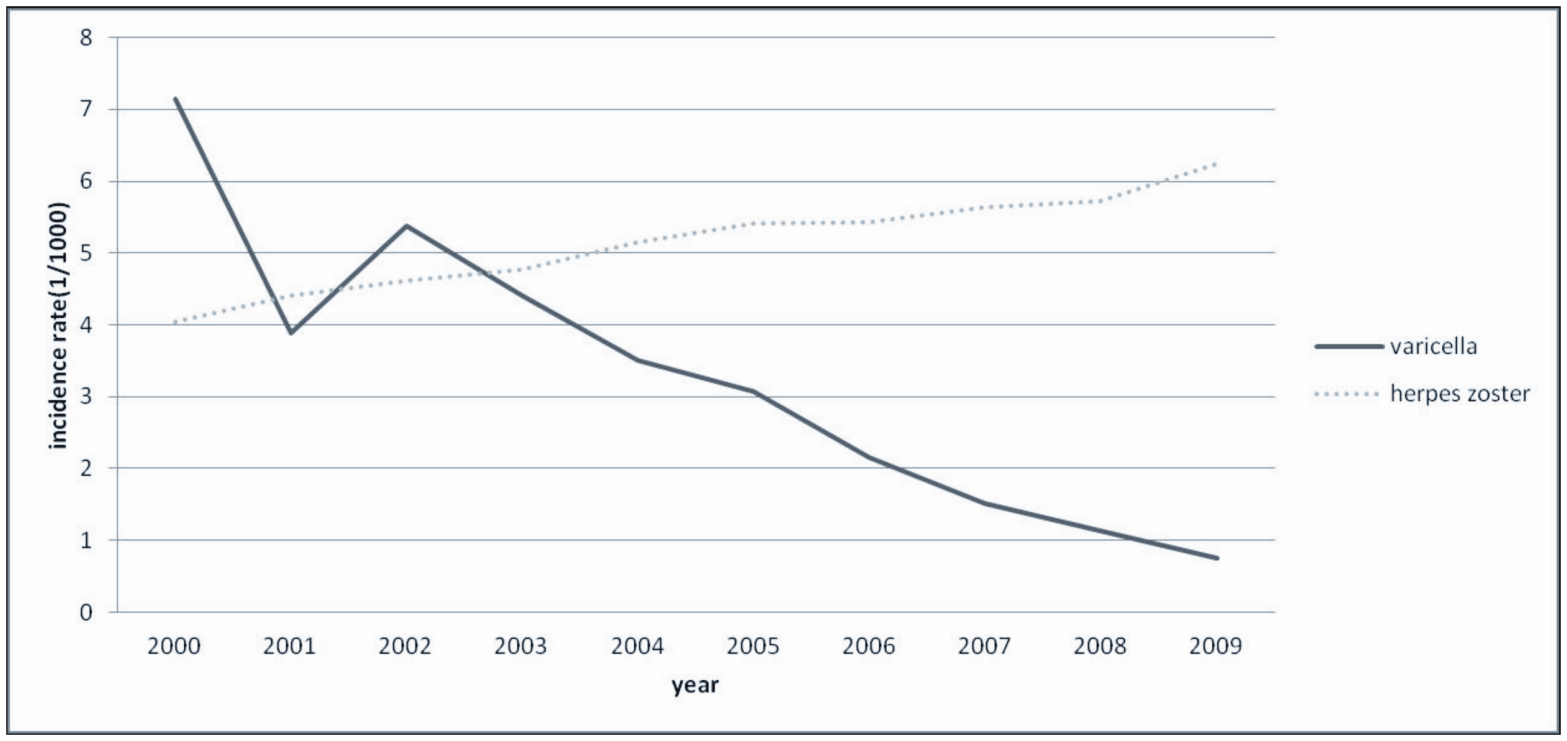
Annual incidence rates of varicella and herpes zoster from 2000 to 2009.

Figure 2 shows the annual incidence of varicella and HZ from 2000 to 2009. The incidence rate of varicella dropped from 7.14 to 0.76 per 1,000 person-years, whereas the incidence of HZ increased steadily from 4.04 to 6.24 per 1,000 person-years.

### Seasonality of Varicella and Herpes Zoster

**Figure 3:**
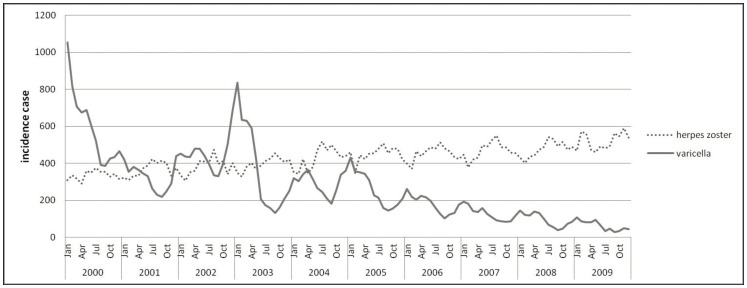
Seasonality of herpes zoster and varicella: annual trend of monthly incident cases of herpes zoster and varicella in 2000–2009.

**Figure 4:**
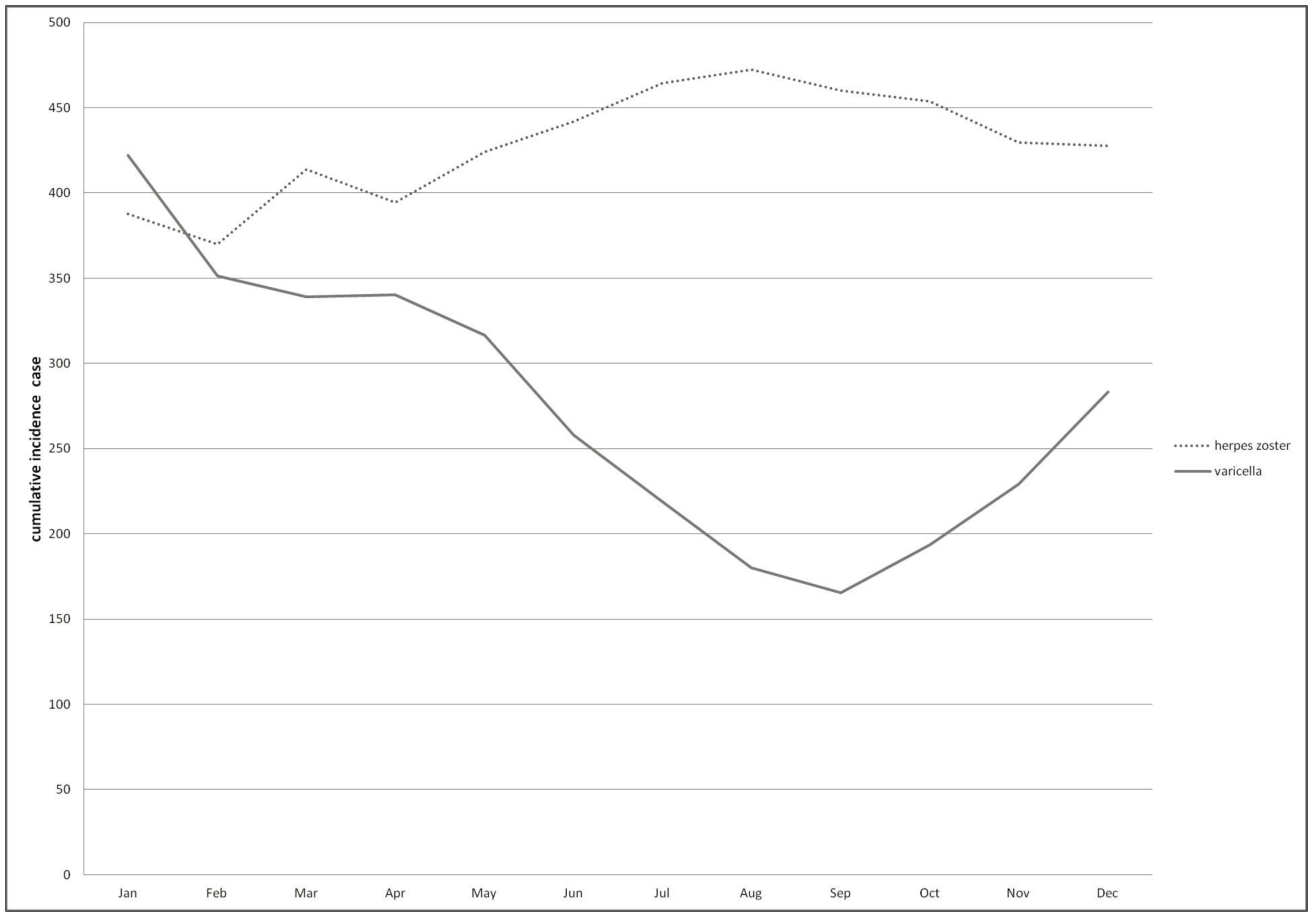
Seasonality of herpes zoster and varicella: monthly average incident cases from 2000 to 2009.

Figure 3 shows the trend of monthly incident cases of varicella and HZ from 2000 to 2009. The slowly increasing trend of HZ became the troughs of declining varicella. The incident cases presented a mirror image between the two diseases. Figure 4 shows the cumulative incidence rates of HZ and varicella stratified by month during the study period. The incidence of HZ peaked in August, whereas that of varicella appeared as a deep concave in September.

### Multivariate Poisson Regression Analysis Results

Poisson regression analysis further measured the relationship between the incidence of HZ and that of varicella during prevaccination (2000–2003) and postvaccination (2006–2009). We excluded the data from 2004 and 2005 to avoid the “wash-out” effect of the official vaccination policy launched in 2004. Vaccination was used as a stratification variable.

Compared with the incidence in 2003, the incidence of HZ in 2000 had a rate ratio (RR) of 0.80 (95% CI 0.77–0.85) (Table 1). 2001 and 2002 had higher RRs (0.88; 95% CI 0.84–0.95) and (0.96; 0.91–1.01), respectively. The situations in 2006, 2007, and 2008 compared with 2009 were similar for the post-vaccination period, with all *p*-values being significant. The seasonal association became statistically insignificant for both periods. Females tended to be at higher risk during both the pre-vaccination (RR = 1.09, 95% CI 1.05–1.13) and post-vaccination periods (RR = 1.13, 95% CI 1.10–1.17).

**Table 1:** Incidence rate ratios of herpes zoster for annual trend, and by season, sex, varicella, temperature and sunny hours, before and after vaccination.

	Before vaccination, 2000–2003				After vaccination, 2006–2009			
Parameter	Rate ratio	Wald 95% CI		*p* value	Rate ratio	Wald 95% CI		*p* value
Year2000; 2006*	0.804	0.765	0.848	<0.0001	0.858	0.821	0.898	<0.0001
Year2001; 2007*	0.884	0.841	0.930	<0.0001	0.887	0.848	0.926	<0.0001
Year2002; 2008*	0.958	0.913	1.006	0.08	0.914	0.875	0.955	<0.0001
Year2003; 2009*	1.0	1.0	1.0	.	1.0	1.0	1.0	.
Spring	0.986	0.930	1.047	0.65	0.972	0.924	1.024	0.29
Autumn	1.044	0.990	1.103	0.11	1.005	0.960	1.052	0.83
Winter	1.038	0.957	1.127	0.36	0.975	0.910	1.047	0.49
Summer	1.0	1.0	1.0	.	1.0	1.0	1.0	.
Female	1.090	1.052	1.130	<0.0001	1.134	1.100	1.170	<0.0001
Male	1.0	1.0	1.0	.	1.0	1.0	1.0	.
Varicella	1.006	0.999	1.013	0.072	1.016	1.002	1.031	0.029
Temperature	1.017	1.011	1.024	<0.0001	1.014	1.009	1.020	<0.0001
Sunny hours	1.021	1.012	1.030	<0.0001	1.017	1.010	1.025	<0.0001

* 2006, 2007, 2008, and 2009 are for after vaccination.

CI, confidence interval.

The effect of temperature was attenuated by 18.5% ≈ (0.0173–0.0141)/0.0173 (data not shown) in association with vaccination. For 1°C and 10°C increments in temperature, the estimated RRs were 1.02 and 1.19, respectively, during the prevaccination period. The same increments resulted in RRs of 1.01 and 1.15 during the postvaccination. For sunny hours, the risk attenuation associated with vaccination was approximately 16.6%≈ (0.0205–0.0171)/0.020.

Table 1 also shows that the RR of varicella with HZ incidence was 1.006 (effect (β) = 0.006, *p*-value = 0.07) during prevaccination. The corresponding RR was 1.02 (effect (β) = 0.016, *p*-value = 0.029) in the post-vaccination period. These results indicated that within a specific area during the postvaccination period, an increase of 10 incident cases of varicella within one week leaded to a RR of 1.016 for HZ, i.e. an increase of one person-week of incident varicella corresponded to 1.6% of increase (1.016–1 = 0.016 = 1.6%) for HZ incidence cases.

By contrast, Table 2 shows that the overall varicella risk decreased yearly in both periods, particularly from 2006 to 2009. The RR in 2001 dropped sharply to 0.829 (β = −0.187, *p*<0.001). Compared with the summer varicella incidence during the prevaccination period, the incidence in spring was significantly higher, with an RR of 1.39 (95% CI 1.30–1.48). The corresponding RR in winter was 1.43 (95% CI 1.31–1.56). The coefficient estimates had higher absolute values (0.4873, −0.02255, and 0.5135) during the postvaccination period than during prevaccination (data not shown). The corresponding RRs were 1.63, 0.80, and 1.67 for spring, fall, and winter, respectively, compared with that for summer. The temperature effect was also attenuated by vaccination, with the parameter estimate increasing from −0.0148 (RR = 0.985) per 1°C increase in pre-vaccination to 0.0076 (RR = 1.008) during postvaccination (*p = *0.1821). The sunny hours also became non-significant during postvaccination, with RR increasing slightly from 1.011 (β = 0.0106, *p = *0.035) in the prevaccination period to 1.012 (β = 0.0116, *p = *0.158) during postvaccination.

**Table 2:** Incidence rate ratios of varicella for annual trend, and by season, sex, varicella, temperature and sunny hours, before and after vaccination.

	Before vaccination, 2000–2003				After vaccination, 2006–2009			
Parameter	Estimate rate ratio	Wald 95% CI		*P* value	Estimate rate ratio	Wald 95% CI		*P* value
Year2000;2006*	1.487	1.408	1.571	<0.0001	2.849	2.565	3.165	<0.0001
Year2001;2007*	0.829	0.780	0.882	<0.0001	1.870	1.674	2.092	<0.0001
Year2002;2008*	1.213	1.147	1.284	<0.0001	1.402	1.247	1.578	<0.0001
Year2003;2009*	1.0	1.0	1.0	.	1.0	1.0	1.0	
Spring	1.387	1.298	1.481	<0.0001	1.627	1.461	1.813	<0.0001
Autumn	0.878	0.821	0.941	0.0002	0.798	0.712	1.117	0.0001
Winter	1.433	1.314	1.564	<0.0001	1.672	1.450	1.927	<0.0001
Summer	1.0	1.0	1.0		1.0	1.0	1.0	.
Female	0.982	0.946	1.021	0.378	1.057	0.990	1.127	0.099
Male	1.0	1.0	1.0		1.0	1.0	1.0	.
Herpes zoster	0.927	0.919	0.935	<0.0001	0.956	0.946	0.967	<0.0001
Temperature	0.985	0.978	0.992	<0.0001	1.008	0.996	1.019	0.182
Sunny hours	1.011	1.001	1.021	0.035	1.012	0.995	1.028	0.158

* 2006, 2007, 2008, and 2009 are for after vaccination.

CI, confidence interval.

Table 2 also shows that HZ was negatively associated with the incidence of varicella, with an RR of 0.927 (β = −0.076, *p*<0.001) for prevaccination and 0.956 (β = −0.045, *p*<0.001) for postvaccination. This finding further indicated that varicella vaccination diminished the association between HZ and the risk of varicella. This was further discussed in the subsequent section.

## Discussion

Several important risk factors have been associated with the incidence of HZ, including age and cell-mediated immunosuppressive disorders [9]. The increased incidence may be due to immunologic senescence caused by aging and an underlying decrease in immunity attributable to the disease itself or immunosuppressive treatment. The endogenous boosting theory has proposed that asymptomatic reactivation induces the anti-VZV cell-medicated immunity, thus varicella vaccination increases the immunity and the incidence of HZ is decreased [1], [2]. However, on the other hand, exogenous boosting theory proposed that contact with children infected with chickenpox reinforces immunity and decreases the incidence of HZ. Vaccination may decrease the amount of environment chickenpox virus amount, which thus increases the incidence of HZ.

Jardine et al. found that the HZ incidence among Australian adults 20 years and older increased from 2% to 6% annually after the implementation of a vaccination program for children [10]. This association, however, is inconclusive. A recent study in Taiwan also reported a 20% increase in the incidence of HZ from 2006 to 2008 compared to that from 2004 to 2005. Vaccination partly explains this trend because the incidence of HZ gradually increased before vaccination [11]. Another recent study in the United States reported no significant difference in the HZ incidence between adults residing in high varicella and low-coverage states [8]. They found no obvious changes in health-seeking behavior and healthcare access among the study populations. Thus, the varicella vaccination program is not the sole factor associated with the increase in HZ incidence.

In our study, the incidence of varicella fluctuated from 2000 to 2003, with a remarkable decline after 2005. Because of high vaccine coverage rate, the varicella vaccination program likely played a role in this change. However, the incidence of HZ gradually increased before and after varicella vaccination, as shown in Figure 3. There is no apparent “turning point” in and after 2004, as shown in the figure.

The Poisson model–estimated effect (β) of HZ in association with varicella before vaccination is 0.006, with an RR of 1.006 (*p* = 0.072), a moderate association. This is because a pilot pediatric varicella immunization has been initiated in limited areas before conducting the vaccination for all children. Therefore, there was a steady increase of HZ even before the vaccination program was fully implemented after 2005 with a mean coverage rate of 90%. This result is consistent with a recent study by Gaillat *et al*. [7]. After the vaccination, the corresponding estimated effect is 0.016 (*p* = 0.029). The incidence of herpes zoster in association with varicella increased 1.6% within one week. Although the increase in HZ incidence associated with the vaccination is statistically significant, this increase does not obstruct the policy of universal vaccination after taking the benefit of significantly decreased incidence of varicella into consideration.

The estimates of varicella incidence associated with HZ were −0.076 (RR = 0.927, *p*<0.0001) before vaccination, and −0.045 (RR = 0.956, *p*<0.0001) after vaccination. This result indicates that HZ is negatively associated with the occurrence of varicella, and the association declines after varicella vaccination. In other words, the incidence of HZ increases slightly, while the incidence of varicella is decreased. VZV may maintain at a constant amount without extinction. To the best of our knowledge, this is the first study demonstrating that there is mutual effect between HZ and varicella.

The precise seasonal relationship between varicella and zoster is still unclear. Primary VZV infections resulting from varicella is seasonal in temperate zones [12]–[14]. However, the seasonality of HZ incidence has not been well studied in other areas [9]. Limited clinical studies have reported an increase in zoster incidence in summer; thus, no seasonal association between varicella and herpes HZ has been inferred. A recent large-scale study in Japan clearly demonstrated the seasonality of HZ, with 75.9% more cases diagnosed in February than in August [15]. Similarly, our study also reveals an obvious seasonality with a mirror effect between these two diseases. However, our statistical models interpret the seasonality after adjusting for other covariates. First, when season is the only climate variable included in the models, both diseases are significantly associated with season (data not shown). However, temperature is the climate variable significantly associated with the incidence of HZ if the model simultaneously accounts for season, temperature, and sunny days. The incidence increases with the temperature. Therefore, the “traditional” seasonal association (peaking during summer) on the increase of zoster is likely due to the changes in temperature (Table 1). Second, with temperature included in the model, the incidence of varicella is also significantly associated with the season. The strong intrinsic seasonality of varicella does not change its seasonality even after vaccination. The difference in seasonality between HZ and varicella is caused by the highly contagious, airborne transmission of varicella. The virus spreads easily through coughing or sneezing from ill individuals or through direct contact with the rash. The season merely functions with its natural effect on VZV activity in the environment. Third, additional sunny hours may increase exposure to ultraviolet irradiation, which may act as an immunosuppressant and reduce immunity to VZV [16]. The association between seasonal climatological factors and varicella and HZ development reflects the importance of public health prevention for potential varicella outbreak when the weather is warm.

No significant gender difference was observed in the incidence of varicella in this study, but women have a higher risk of suffering from HZ than men. This finding is consistent with clinical observations regarding VZV. The high incidence of HZ among elderly women in this study is also consistent with previous studies [15], [17], [18]. Highly contagious varicella may explain why children have a similar risk of infection, but no clear explanation why women have a higher risk of infection than men.

This study used a representative large cohort with longitudinal data, allowing the observation of a chronological trend between varicella in children and HZ in adults. This chronological trend observed for one million people has effectively represented the association in the general population. However, our study is not without limitations. First, immunosuppression is a confounding factor that we were unable to control for lack of information in our database. However, Taiwan is an area with a low HIV prevalence of 0.16%, mostly in subjects younger than our herpes group. There were 150 HIV prevalent cases identified in the claims data used for this study. Thus HIV infection is too rare to affect HZ. Second, patients who failed to seek medical advice were not included in the insurance claims database. Thus, the annual incidence of these diseases may be slightly underestimated in this study, but not to the extent that it decreased its reliability in estimating the relationships among HZ, varicella, weather and season. Furthermore, because the national insurance system covers approximately 99% of the population, our data set is similar to the entire insured population in terms of sex and age distribution. Thus, biases caused by the sampling procedure are unlikely.

Third, Our data included no pre-vaccination information. The association between the rising incidence of HZ and the decreasing incidence of VZV observed in this study may be suspected as a coincidence. The vaccination program against varicella started in 2000 within a small area in Taiwan, as a pilot program before the health authority officially launched a full-fledged vaccination program for all children in 2004. The trends show that VZV vaccination might be in part responsible for the observed increase in HZ incidence. Fourth, the study period might be not long enough to finalize the shifting incidence patterns of the two diseases. There are some children among whom varicella may still be circulating. But, we were able to observe the increasing incidence of HZ, while the incidence of varicella infection was declining. Again, this limitation does not invalidate our measurement of the relationship between HZ and varicella, as well as other factors. It is possible that as more individuals become immune by vaccination, the booster effect on the protection against HZ due to the contact with active varicella cases becomes increasingly less likely. Our further data analysis for the annual trend of age-specific incidence of HZ infection showed that teenagers also presented with HZ infection, increasing with time from 358 cases in 2000 to 421 cases in 2009 (data not shown). A previous report suggested that for neutralization of herpes viridae anti-gC antibodies are particularly important [19] whereas at the same time antibody titers against gC (ORF14) have been reported to be lower in vaccinees compared to children after wild type infection [20]. This might possibly explain at least in part the rising incidence of HZ in both children and adults. However, The increasing trend is greater in adults than in children.

In conclusion, varicella vaccination may be partially associated with the increase in the incidence of HZ in Taiwan. Vaccination affects the prevention of varicella and may lessen its relationship with temperature and UV exposure. However, seasonal variations remain statistically significant. HZ has also a strong association with temperature and UV. The climate association also deserves further study.
